# Towards a universal definition of postpartum hemorrhage: retrospective analysis of Chinese women after vaginal delivery or cesarean section: A case-control study

**DOI:** 10.1097/MD.0000000000021714

**Published:** 2020-08-14

**Authors:** Qiang Wei, Yi Xu, Li Zhang

**Affiliations:** Department of Obstetrics and Gynecology, Key Laboratory of Birth Defects and Related Diseases of Women and Children of the Ministry of Education, West China Second University Hospital, Sichuan University, Chengdu, PR China.

**Keywords:** postpartum hemorrhage, delivery, obstetric, cesarean section

## Abstract

Postpartum hemorrhage (PPH) is a leading cause of maternal morbidity and mortality, yet it is inconsistently defined, preventing accurate estimation of its incidence and identification of risk factors. Here we began to explore a unified definition of PPH that may be valid for vaginal delivery and cesarean section.

Medical records of women who underwent vaginal delivery or cesarean section at our tertiary medical center between January and December 2018 were retrospectively analyzed. Patients who delivered by each route were compared in terms of PPH incidence and risk factors depending on different blood loss cut-off values.

A total of 560 vaginal deliveries and 393 cesarean sections were analyzed. Vaginal deliveries were associated with significantly greater blood loss based on change of hemoglobin level, but significantly lower blood loss based on clinical estimation. When PPH was defined as blood loss ≥500 ml based on change of hemoglobin level, its incidence was 57.7% for vaginal deliveries and 28.2% for cesarean sections. The corresponding incidences were 15.4% and 3.3% when PPH was defined as blood loss ≥1000 ml based on change of hemoglobin levels. Independent risk factors for PPH in vaginal deliveries were lateral perineotomy (OR 2.835, 95%CI 1.694-4.743), suturing by a junior physician (OR 3.456, 95%CI 2.005-5.956), and long time from delivery of placenta to return to the recovery room (OR 1.013, 95%CI 1.003-1.022). A risk factor for PPH in cesarean sections was a long time from delivery of the fetus until the end of the operation.

PPH is a significantly underestimated obstetric problem, especially in vaginal deliveries. Regardless of delivery route, hemoglobin-based blood loss of 500 ml and 1000 ml may be useful, respectively, as early warning and diagnostic cut-off values.

## 1 Introduction

Postpartum hemorrhage (PPH) is a leading cause of maternal morbidity and mortality. It remains the leading direct obstetric cause of maternal death, accounting globally for around one quarter of maternal deaths and severe adverse maternal outcomes, as well as for more than 80,000 maternal deaths in 2015 alone.^[1–3]^

One of the obstacles to understanding PPH incidence and risk factors is the lack of a universal definition. Guidelines from the World Health Organization, Royal College of Obstetricians and Gynecologists and French College of Gynecologists and Obstetricians define PPH as the loss of at least 500 ml of blood from the genital tract within 24 hours of birth, regardless of whether the delivery was vaginal delivery or cesarean section.^[4–6]^ In contrast, guidelines from the International Federation of Gynecology and Obstetrics and the Chinese Ministry of Health and Queensland Health Authority define PPH as loss of at least 500 ml in a vaginal birth or 1000 ml in a cesarean section.^[7–9]^ The American College of Obstetricians and Gynecologists defines PPH as cumulative loss of at least 1000 ml of blood, or any amount of blood loss if accompanied by signs or symptoms of hypovolemia, within 24 hours after birth.^[10]^ Thus, many official bodies apply higher blood loss cut-offs to cesarean section than to vaginal delivery.

Over the last several years, our clinical experience as a large tertiary-care medical center in China has highlighted the potential inadequacy of early PPH recognition and diagnosis. We began to notice that vaginal deliveries tended to lead to greater postpartum blood loss than cesarean sections, and that blood loss based on change of hemoglobin levels deviated substantially from loss estimated by midwives or doctors during delivery, especially vaginal deliveries. In vaginal delivery, especially in patients with blood loss more than 1000 ml, the amount of bleeding can be significantly underestimated.

In an effort to develop a more uniform definition of PPH that may be appropriate for our patients regardless of delivery route, we retrospectively analyzed medical records for pregnancies at our hospital. We examined PPH incidence based on different cut-off values for blood loss and delivery route, and we explored PPH risk factors for each route.

## 2 Methods

### 2.1 Study population

Medical records were retrospectively analyzed for pregnancies between January and December 2018 in the Department of Obstetrics at West China Second Hospital of Sichuan University. The study protocol was approved by the Ethics Committee of West China Second Hospital of Sichuan University.

Women were enrolled in the study only if they had received standard prenatal care, and if complete blood analysis was available within 72 hours before delivery as well as 24 to 72 hours afterward. We recruited patients treated by only one of the 5 medical teams in our obstetrics department in order to avoid confounding due to differences in clinician practice and experience. Women were classified according to whether they underwent vaginal delivery or cesarean section.

Women were excluded from the study if they had

(1) severe internal or surgical complications, such as heart, hematological, liver or kidney disease;
(2) pernicious placenta previa; or
(3) hypertensive disorder during pregnancy (systemic vasospasm of small blood vessels, blood concentration, or abnormally small increase in blood volume in the third trimester). Women were also excluded if they
(4) received blood transfusion before delivery.

### 2.2 Data collection

Demographic and clinical data on study subjects were collected retrospectively on numerous variables, including demographic data, obstetric specialty, delivery route, labor stage, neonate birth weight, blood loss of clinical estimation, hemoglobin levels before and after delivery, and factors that may be linked to PPH or pregnancy-related complications.

### 2.3 Data collection of blood loss

Blood loss was collected with 2 methods. One is clinically estimated blood loss, the other is actual blood loss which was calculated by the change of hemoglobin levels.

Clinically estimated blood loss was based on clinical estimation and obtained from surgical and nursing records. Blood loss during vaginal delivery was estimated visually by the midwife and weighing from the total amount of blood on gauze, perineal pads and cloth sheet. The amount of blood lost during cesarean section was estimated visually by the chief surgeon and weighing from the total amount of blood on gauze, gauze pads and cloth sheet during the operation, after removing amniotic fluid. After the patient returned to the ward from the delivery room or operating room, blood loss during the first 24 hours postpartum was determined from record of the ward nurse by weighting the blood on perineal pads.

Actual blood loss was calculated from the change in hemoglobin levels^[11]^: each decrease of 10 g/L of hemoglobin was taken to indicate loss of 400 ml.^[9]^

### 2.4 Statistical analysis

Data were analyzed using SPSS 24.0 (IBM, Armonk, NY). Data showing normal distribution and homogeneous variance were reported as mean ± SD and compared between two groups using the independent-samples *t* test. Otherwise, data were reported as the median and interquartile range, and compared between two groups using the nonparametric rank sum test. Differences in composition and rates of variables were assessed for significance using the chi-squared test. Multivariate analysis using logistic regression was conducted to identify potential PPH risk factors. Differences were considered significant if associated with *P* < .05.

## 3 Results

### 3.1 Characteristics of the study population

We recruited 621 women who delivered vaginally and 674 who delivered by cesarean section during the enrollment period. A total of 61 women in the vaginal delivery group and 281 in the cesarean section group showed no postpartum decrease in hemoglobin, so their blood loss could not be assessed based on hemoglobin change, and they were therefore excluded from analysis. The remaining 560 women with vaginal deliveries and 393 with cesarean sections were included in the analysis (Table 1). In terms of characteristics, maternal age, gravidity, parity, and birthweight in the cesarean section group were higher than those in the vaginal delivery group, gestational age in the cesarean section group was lower than that in the vaginal delivery group, all with statistically significant differences. In terms of obstetric complications, placenta previa, placenta adhesion, gestational diabetes mellitus, intrahepatic cholestasis of pregnancy and hyperdistention of uterus (polyhydramnios, macrosomia, and twin pregnancy) in the cesarean section group were higher than those in the vaginal delivery group, with statistically significant differences. The difference between the rate of preterm labor and placenta abruption in the 2 groups was no statistically significant.

**Table 1:**
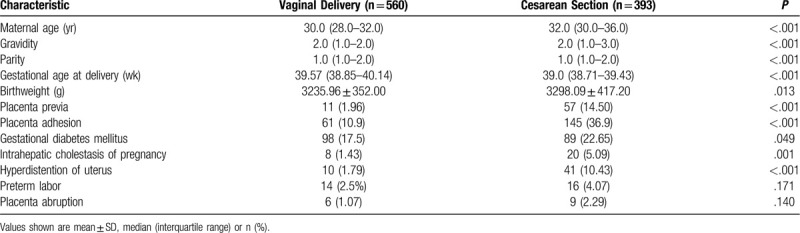
Comparison of demographic and clinical characteristics between the 2 groups.

### 3.2 Actual and clinically estimated blood loss in the first 24 hours after delivery

Actual postpartum blood loss, calculated based on the pre- and postpartum difference in hemoglobin levels, was higher in the vaginal delivery group [median (interquartile range), 560.0 (320.0-800.0) ml] than in the cesarean section group [320.0 (160.0-520.0) ml, *P* < .001). Conversely, clinically estimated postpartum blood loss was significantly smaller in the vaginal delivery group [320.0 (240.0-380.0) ml vs 350.0 (300.0-400.0) ml, *P* < .001). In the vaginal delivery group, actual blood loss was significantly higher than the clinically estimated loss, whereas the converse was true in the cesarean section group (Table 2).

**Table 2:**
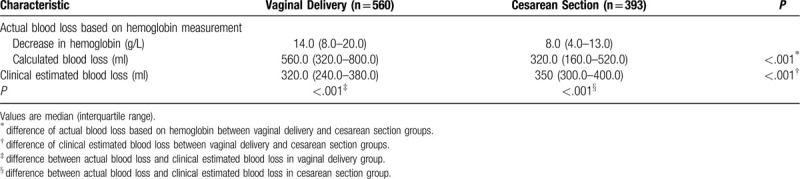
Comparison of blood loss between vaginal delivery and cesarean section.

### 3.3 PPH incidence based on different diagnostic criteria

According to the definition of PPH as blood loss of at least 500 ml within 24 hours after birth, PPH rate based on change of hemoglobin levels was significantly higher in the vaginal delivery group (323/560, 57.7%) than in the cesarean section group (111/393, 28.2%) (Table 3). In contrast, PPH rate based on clinical estimation was similar between the vaginal delivery and cesarean section groups (10.4% vs 13.0%). According to the definition of PPH as blood loss of at least 1000 ml within 24 hours after birth, PPH rate based on change of hemoglobin levels was significantly higher in the vaginal delivery group (86/560, 15.4%) than in the cesarean section group (13/393, 3.3%) (Table 3). In contrast, PPH rate based on clinical estimation was similar between the two groups (1.6% vs 1.0%). So increasing the cut-off from 500 to 1000 ml led to the same relative relationships between the 2 groups and between calculations based on hemoglobin levels or clinical estimation.

**Table 3:**
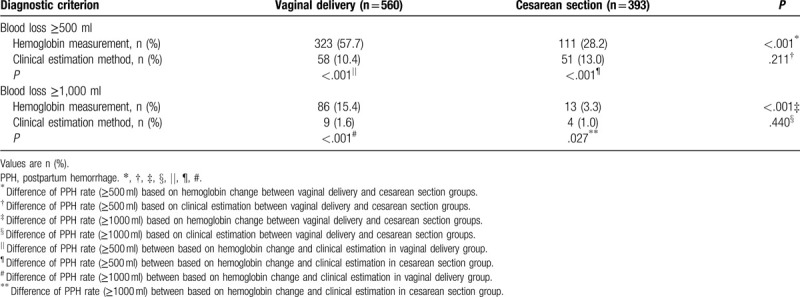
Comparison of postpartum hemorrhage rate between vaginal delivery and cesarean section.

### 3.4 PPH risk factors in vaginal deliveries

We subdivided the vaginal delivery group into those who experienced PPH (n = 323) and those who did not (n = 237), based on whether the decrease in hemoglobin indicated a blood loss of at least 500 ml. The PPH subgroup showed significantly longer duration of the second labor stage and longer interval from delivery of placenta to return to the recovery room, and significantly higher rates of lateral perineotomy, primiparity, midwifery and suturing by a junior clinician (Table 4). Logistic regression was performed in which the variables were significant differences in the above univariate analysis, in order to avoid confounding bias. This regression identified independent PPH risk factors to be lateral perineotomy, suturing by a junior clinician, and interval from delivery of the placenta until return to the recovery room (Table 5).

**Table 4:**
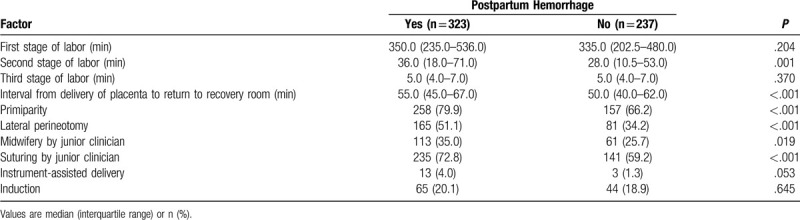
Identification of factors associated with postpartum hemorrhage in vaginal delivery.

**Table 5:**
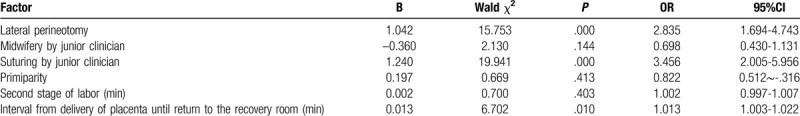
Logistic regression to identify predictors of postpartum hemorrhage in vaginal delivery.

### 3.5 PPH risk factors in cesarean sections

Using the same criterion as in the vaginal delivery group, the cesarean section group was also subdivided into those who experienced PPH (n = 111) and those who did not (n = 282). The PPH group showed significantly longer interval from delivery of the fetus until the end of surgery, but otherwise no other significant differences (Table 6). Since Logistic regression analysis is not possible, thus we were unable to identify independent risk factors of PPH for cesarean section in our cohort.

**Table 6:**
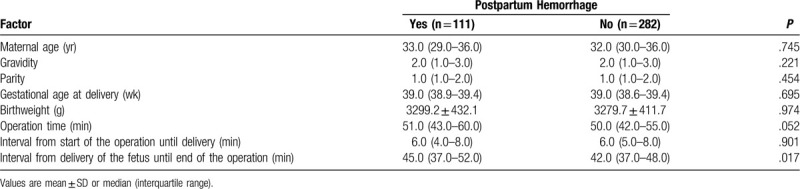
Identification of factors associated with postpartum hemorrhage in cesarean section.

## 4 Discussion

In our study, we found that clinical estimation underestimated blood loss for vaginal deliveries, whereas it overestimated the loss for cesarean sections. Pregnancy complications that increase the incidence of PPH were higher among cesarean sections than vaginal deliveries. Nevertheless, the incidence of PPH was significantly higher in vaginal deliveries than in cesarean sections, regardless of whether the diagnostic threshold was 500 or 1000 ml based on change of hemoglobin level. These results suggest that the standard practice of applying a higher blood loss cut-off to cesarean sections than vaginal deliveries is inappropriate for modern clinical practice. A unified criterion regardless of delivery route may be needed.

### 4.1 Inadequacy of clinical estimation of blood loss during delivery

Most deaths resulting from PPH occur during the first 24 hours after birth and could be avoided through timely and appropriate management, such as prophylactic uterotonics during the third stage of labor.^[4]^ Underestimating blood loss can delay diagnosis of PPH and thereby prevent timely management.^[9]^ Overestimating blood loss can lead to unnecessary use of blood products and medications and expose patients to possible complications.^[12]^ We found that clinical estimation using visual and weighing methods underestimated blood loss for vaginal deliveries, whereas it overestimated the loss for cesarean sections. These inaccuracies had correspondingly large effects on PPH incidence, as shown in previous studies.^[13,14]^ In fact, clinical estimation may become increasingly inaccurate as blood losses increase.^[15]^

The present study shows that clinical estimation of blood loss is not accurate, especially in vaginal deliveries. This may depend on staff ability, volume and speed of blood loss, and bias based on the current criteria for diagnosing PPH. Our results suggest the need to improve staff ability to estimate blood loss by relying on more objective methods that accurately assess the full loss in vaginal deliveries.

### 4.2 Proposed cut-off blood loss values for PPH early warning and diagnosis

Whether vaginal delivery or cesarean section is associated with greater postpartum blood loss is controversial.^[4–10]^ Regardless of whether we selected a blood loss cut-off of 500 or 1000 ml, PPH incidence was higher among women with vaginal deliveries than among those with cesarean sections. Although our cesarean section subgroup included many women with PPH risk factors, we obtained similar results as several studies that included primarily women at low PPH risk in the cesarean section group.^[16]^ The higher blood loss after vaginal delivery in our study may reflect that cesarean sections at our hospital are performed by more senior clinicians, the procedure is shorter and more controlled than vaginal delivery, blood loss is easier to track and estimate during cesarean section, and staff may be less prepared to suspect PPH in vaginal deliveries because of the commonly held idea that cesarean sections involve greater blood loss.

Whatever the cause of the blood loss difference between the vaginal delivery and cesarean section in our study, our results imply that current guidelines stipulating a higher blood loss cut-off for cesarean section may not be the most appropriate. Our study showed a PPH incidence of 15.4% for vaginal deliveries and 3.3% for cesarean sections using a cut-off of 1000 ml. Thus, 1000 ml may be a reasonable cut-off to apply for diagnosing PPH in women, regardless of delivery route.

Since early recognition of PPH and rapid intervention are essential for good prognosis,^[17,18]^ many clinical guidelines include vital signs in the diagnosis of PPH.^[7,8,10,19]^ However, these signs of hemodynamic instability occur late and can be masked during pregnancy.^[20]^ We found that a blood loss cut-off value of 500 ml led to excessively high PPH incidence among vaginal deliveries (57.7%) and cesarean sections (28.2%), so this may be a useful “early warning” cut-off value in the clinic.

### 4.3 PPH risk factors in vaginal delivery and cesarean section

In the cesarean section group, PPH was associated with longer interval from delivery of the fetus until the end of the operation. In the vaginal delivery group, logistic regression showed that PPH was associated with lateral perineotomy, suturing by a junior clinician and longer interval from delivery of the placenta until return to the recovery room. Previous work has shown that perineotomy is a risk factor for PPH.^[6]^ Our finding of longer interval from delivery until return to the recovery room as a risk factor is consistent with the idea that longer suturing time (such as because junior clinicians have less experience than senior midwives) can lead to continuous, slow bleeding from the incision. Our results highlight the need for strengthening suturing ability and for making careful clinical decisions (e.g., about perineotomy) during delivery.

In this study, 281 patients (41.7%) in the cesarean section group and 61 patients (9.8%) in the vaginal delivery group showed no decrease in postpartum hemoglobin. Maybe because the blood loss of these women was too little that their hemoglobin levels were negligibly affected by the blood loss. The proportion in cesarean section group with no decrease in postpartum hemoglobin was much higher than that in vaginal delivery group, which further showed that postpartum hemorrhage in cesarean section group was significantly less than that in vaginal delivery group. If we identify the patients with hemoglobin not dropped as blood loss less than 500 ml, the PPH rate in vaginal delivery is also more higher than in cesarean section. According to blood loss of at least 500 ml within 24 hours after birth, PPH rate was significantly higher in the vaginal delivery group (323/621, 52.0%) than in the cesarean section group (111/674, 16.5%). According to blood loss of at least 1000 ml within 24 hours after birth, PPH rate was also significantly higher in the vaginal delivery group (86/621, 13.8%) than in the cesarean section group (13/674, 1.9%). So whether we excluded women with no decrease in postpartum hemoglobin from analysis or not, the trend of PPH rate in the two groups was same.

Our results should be verified in larger prospective studies.

## 5 Conclusion

Our results suggest the need to re-assess current PPH diagnostic guidelines, and we propose a blood loss cut-off of 500 ml as an early warning indicator and 1000 ml as a diagnostic criterion, irrespective of delivery route.

## Author contributions

Qiang Wei drafted the manuscript, collected and analyzed the data. Li Zhang conceived and designed the study, helped draft the manuscript, revised it critically for important intellectual content, and coordinated data collection. Yi Xu helped with coordination of data collection and analysis, and helped draft the manuscript. All authors read and approved the final manuscript.

**Conceptualization:** Li Zhang.

**Data curation:** Qiang Wei, Yi Xu.

**Formal analysis:** Li Zhang.

**Methodology:** Li Zhang.

**Supervision:** Li Zhang.

**Writing – original draft:** Qiang Wei, Yi Xu.

**Writing – review & editing:** Li Zhang.
